# Prickle2 and Igsf9b Coordinately Regulate the Cytoarchitecture of the Axon Initial Segment

**DOI:** 10.1247/csf.20028

**Published:** 2020-07-08

**Authors:** Md. Imrul Hasan Chowdhury, Tomoki Nishioka, Noriko Mishima, Toshihisa Ohtsuka, Kozo Kaibuchi, Daisuke Tsuboi

**Affiliations:** 1 Department of Cell Pharmacology, Nagoya University, 65 Tsurumai-cho, Showa, Nagoya, Aichi 466-8550, Japan; 2 Institute for Comprehensive Medical Science, Fujita Health University, Toyoake, Aichi 470-1192, Japan; 3 Department of Biochemistry, Yamanashi University, 1110 Shimokato-cho, Yamanashi 409-3893, Japan

**Keywords:** Prickle2, Igsf9b, axon initial segment, neuronal excitability, ASD

## Abstract

*Prickle2* has been identified in genetic studies of subjects with autism spectrum disorder (ASD) and epilepsy, but the pathological mechanism of Prickle2 remains to be fully understood. Proteomic analysis of Prickle2 with mass spectrometry revealed twenty-eight Prickle2 interactors, including immunoglobulin superfamily member 9b (Igsf9b), in the brain. Here, because Igsf9 family proteins are associated with psychiatric diseases and seizures, we studied the physiological interaction between Prickle2 and Igsf9b. Prickle2 colocalized with Igsf9b in cultured hippocampal neurons. Knockdown of Prickle2 affected the subcellular localization of Igsf9b. Interestingly, Igsf9b localized along axonal processes in a pattern opposite to the ASD-related molecule ANK3/AnkG. AnkG is a major component of the axon initial segment (AIS), where a variety of ASD and epilepsy susceptibility proteins accumulate. Igsf9b-knockdown neurons displayed altered AnkG localization. Prickle2 depletion caused defects in AnkG and voltage-gated Na+ channel localization, resulting in altered network activity. These results support the idea that Prickle2 regulates AnkG distribution by controlling the proper localization of Igsf9b. The novel function of Prickle2 in AIS cytoarchitecture provides new insights into the shared pathology of ASD and epilepsy.

## Introduction

Autism spectrum disorder (ASD) is a neurodevelopmental disorder marked by several abnormal behaviors: deficits in communication and social interaction and repetitive movements. These symptoms begin in early childhood and persistently interfere with daily life throughout life. Many physical and mental health conditions, such as epilepsy, gastrointestinal symptoms, sleep disturbance, and feeding problems, co-occur with ASD (Tye *et al.*, 2018). In particular, ASD and epilepsy frequently coexist (Lukmanji *et al.*, 2019). Both diseases share comorbid symptoms, such as altered electroencephalogram and imbalance of the ratio of excitatory and inhibitory activity. This clinical and physiological overlap leads us to speculate a common dysfunction in the modulation of neural network.

Genetic studies of rare nucleotide variants, copy-number variations, and chromosomal abnormalities in ASD have contributed to the identification of various susceptibility genes (Sener *et al.*, 2016). Among the risk factors for ASD, *Prickle2* has been identified in genetic studies of ASD subjects with epilepsy. A chromosomal microdeletion encompassing Prickle2 is associated with ASD and epilepsy (Okumura *et al.*, 2014). A whole-genome exome study found genetic variations of *Prickle2* in patients with these illnesses (Sowers *et al.*, 2013; Tao *et al.*, 2011). These findings suggest that Prickle2 hypofunction is involved in the shared pathology of ASD and epilepsy. However, the pathological mechanism of Prickle2 remains uncertain.

*Prickle* (*Pk*) was originally identified as a core component of planar cell polarity signaling in *Drosophila melanogaster* (Gubb *et al.*, 1999). A *Drosophila Pk*^sple^ mutant lacking an isoform of Pk showed dysregulated vesicle transport to axons, causing severe seizures (Ehaideb *et al.*, 2014). In the mammalian genome, the *Prickle* genes, Prickle1, Prickle2, Prickle3, and Prickle4, are encoded as a protein family and share LIM domains. Mouse Prickle2 is highly expressed in the central nervous system. At the subcellular level, Prickle2 localizes to the postsynaptic density and physically interacts with postsynaptic density protein 95 and NMDA receptors (Hida *et al.*, 2011). Prickle2 at postsynapses controls spine connection by modulating the N-cadherin/Vangl2 interaction (Nagaoka *et al.*, 2014). In a genetic animal model study, Prickle2-knockout mouse shows increased sensitivity to seizures and autism-related behaviors (Sowers *et al.*, 2013). However, the molecular mechanism of Prickle2 underlying the pathology of ASD and seizures remains to be completely uncovered.

The present study was conducted to investigate the pathological mechanism of Prickle2 in neurological diseases. Proteomic analysis of Prickle2 revealed many Prickle2-interacting molecules, including Immunoglobulin superfamily member 9b (Igsf9b). Knockdown studies demonstrated that Prickle2 and its partner, Igsf9b, are involved in positioning of the axon initial segment (AIS), where many ASD and epilepsy susceptibility proteins accumulate.

## Materials and Methods

### DNA constructs

Full-length mouse Prickle2 cDNA (PK2-Full, amino acids (aa) 1–886) was amplified from a mouse cDNA library and subcloned into pCAGGS-HA and pEGFP-C1 (Clontech Laboratories, Palo Alto, CA). To visualize cell morphology, the pBact-mRFP (a modified vector from pBact-myc, Addgene, #87060) vector was employed. The Prickle2 fragments PK2-N1 (aa 1–324), PK2-N2 (aa 1–599), PK2-C1 (aa 600–886), and del325_370 (in which aa 325–370 were deleted) were inserted into pEGFP-C1 and pCAGGS-HA. Full-length Igsf9b cDNA was cloned from the FANTOM cDNA library (DNAFORM, Yokohama, Japan) and then inserted into pFUGW without GFP cDNA (Addgene, #14883). In the knockdown experiments, we used short hairpin RNA (shRNA) vectors and small interfering RNA (siRNA) for cell biological and electrophysiological studies, respectively. The following sequences were used to downregulate Prickle2: 5'-ATGGACAGAATAAATGGAC-3' for scramble, 5'-GCTGGAGAGAAGTTGCGAA-3' for Prickle2, and 5'-GGACCCTACTTCACGGAGT-3' for Prickle2#2. For Igsf9b knockdown, we used an efficient shRNA sequence (5'-CATCAAGTTTGGCTACTAT-3') validated in a previous study (Woo *et al.*, 2013). We cloned these 19 nucleotides into the pSUPER (OligoEngine, MA, USA) RNAi vector. For the rescue experiment in Prickle2-knockdown neurons, we constructed an RNAi-resistant Prickle2 mutant (PK2rs) containing two mutations in the target sequence of the sh-PK2 RNAi vector.

### Analyses by immunoprecipitation and mass spectrometry

The whole brains of mice at postnatal day 9 (PD9) and adult mice were lysed in radioimmunoprecipitation assay (RIPA) buffer (150 mM NaCl, 0.1% Triton X-100, 0.5% sodium deoxycholate, 0.1% SDS (sodium dodecyl sulfate), and 50 mM Tris–HCl, pH 8.0) with protease inhibitors (Roche, MA, USA). The brain extract was immunoprecipitated with the validated anti-Prickle2 antibody (Hida *et al.*, 2011). Mass spectrometry was performed as previously described with some modifications (Nishioka *et al.*, 2012). Briefly, the coprecipitated proteins were extracted from the beads using a guanidine solution (7 M guanidine and 50 mM Tris), reduced via incubation in 5 mM dithiothreitol for 30 min, and alkylated using 10 mM iodoacetamide for 1 h in the dark. The proteins were demineralized, concentrated via methanol/chloroform precipitation, and digested with trypsin (50 mM NH4HCO3, 1.2 M urea, and 0.5 μg of trypsin). A MonoCap C18 Trap Column (50 mm×0.2 mm, AMR, Tokyo Japan) was used to concentrate the sample peptides. The peptides were separated using a 70-min gradient of 5%–40% acetonitrile containing 0.1% formic acid at a flow rate of 400 nL/min. Nanoelectrospray tandem mass analysis was performed using LTQ Orbitrap Fusion mass spectrometry (Thermo Scientific, MA, USA) as described (Nagai *et al.*, 2016). A peak list was generated and calibrated using MASCOT software (Matrix Science, Boston, MA). False discovery rates (FDRs) at the peptide, protein, and site levels were set to 0.01. The candidate substrates were defined as molecules that displayed more than 3-fold increase in the number of identified peptides compared to those in the controls. To validate the binding partners of Prickle2 in the immunoprecipitation assay, we used the following specific antibodies: anti-Prickle2 antibody (pAb, homemade, Hida *et al.*, 2011), anti-Igsf9b antibody (pAb, Cat#: HPA010802, RRID:AB_1079194, Sigma-Aldrich), anti-Igsf9b antibody (clone 993107, Cat#: MAP9140, R&D Systems, MN, USA), and anti-Neuroligin2 antibody (pAb, Cat#: ANR-036, RRID: AB_2341007, Alomone Labs, Jerusalem, Israel).

### Animals

All of the animal studies were conducted in accordance with guidelines from the Animal Care and Use Committee of Nagoya University in Japan and, along with the exclusion criteria, were approved by the Nagoya University Animal Care Committee, Nagoya, Japan (approval number: 31201). Pregnant C57BL/6J mice were purchased from SLC Japan, Inc. (Cat# C57BL/6JJmsSlc female, Shizuoka, Japan) and used for all experiments. The mice were housed in sterile plastic cages (3 mice per cage). The number of mice used is shown in the figures corresponding to each experiment. All efforts were made to minimize animal suffering. In particular, the mice were sacrificed by CO_2_ asphyxiation followed by cervical dislocation. Experimentation was conducted according to the ARRIVE guidelines.

### Cell transfection

Hippocampal neurons were prepared from E17 embryos of wild-type (C57BL/6J) mice as previously described (Taya *et al.*, 2007). Briefly, neurons were seeded onto a poly-D-lysine-coated 35-mm glass-bottom dish and cultured in neurobasal medium (Invitrogen) supplemented with B-27 (Thermo Fisher Scientific, Waltham, MA, USA) and 0.5 mM L-glutamine. The cultured neurons were transfected with Lipofectamine LTX (Thermo Fisher Scientific) and a CalPhos transfection kit (Thermo Fisher Scientific). For the immunoprecipitation assay in HEK293 (ATCC, #CRL1573, VA, USA) cells transiently expressing GFP-Prickle2 and Igsf9b, we precipitated Igsf9b with anti-Igsf9b antibody (RRID: AB_1079194), after which the precipitated proteins were subjected to immunoblotting with anti-GFP antibody (1E4, Cat# M048, RRID: AB_591823, MBL, Nagoya, Japan).

### Immunofluorescence study

We immunostained cultured hippocampal neurons as previously described (Arimura *et al.*, 2009). Briefly, hippocampal neurons were fixed with 4% paraformaldehyde (PFA) in phosphate-buffered saline (PBS) for 15 min and permeabilized with PBS containing 0.2% Triton X-100 for 10 min. For the immunofluorescence study of AnkG, we permeabilized the neurons with 0.2% Triton X-100 for 1 h, followed by blocking with normal serum. Alternatively, for the double immunofluorescence study of AnkG and Igsf9b or NCAM, we employed digitonin permeabilization (0.02% digitonin in PBS) in living neurons to allow the release of small cytosolic components (Werneburg *et al.*, 2002). The fixed neurons were incubated overnight at 4°C with primary antibodies, washed, and incubated for 1 h with secondary antibodies. Fluorescent images were taken with a laser-scanning confocal microscope (LSM780, Carl Zeiss, Oberkochen, Germany). The involvement of Prickle2 in the subcellular localization of Igsf9b was evaluated according to the fluorescence intensities of Igsf9b in wild-type and Prickle2-knockdown neurons. We first measured the integrated density, which is the product of the area of the region of interest (ROI) and mean fluorescence intensity of Igsf9b, and then calculated background-corrected fluorescence intensity in the ROI (integrated density—(area of the selected ROI×the mean fluorescence of background readings). The corrected fluorescence intensity of Igsf9b in each ROI was normalized with the fluorescence intensity of RFP in the same ROI. Data are given as the mean±SE of the relative Igsf9b signal per knockdown cell, with the signal in control cells defined as 1.0.

The following antibodies were used in this study: anti-RFP (5F8, RRID: AB_2336064, ChromoTek, Martinsried, Germany), anti-Tau-1 (PC1C6, Cat# MAB3420, RRID: AB_94855, Millipore, NY, USA), anti-MAP2 (AP-20, Cat# ab11268, RRID: AB_297886, Abcam, NY, USA), anti-Prickle2 (pAb, homemade, Hida *et al.*, 2011), anti-Igsf9b (993107, Cat#: MAB9140, RRID: not determined, R&D Systems, MN, USA), anti-NCAM, (ERIC-1, Cat#: ab6123, RRID: AB_2149537, Abcam), anti-AnkyrinG (N106/36, Cat#: MABN466, RRID: AB_2749806, Sigma-Aldrich), anti-pan-Nav (pAb, Cat#: ASC003, RRID: AB_2040204, Alomone Labs), anti-Bip (40/BiP, Cat#: 610978, RRID: AB_398291, BD Biosciences, Tokyo, Japan), anti-LAMP1 (H4A3, RRID: Cat#: ab25630, AB_470708, Abcam, MA, USA) and anti-Rab8 (4/Rab8, Cat# 610845, RRID: AB_398164, BD Biosciences).

### Multielectrode array

A multisite recording system with a sampling frequency of 20 kHz (MED64, Alpha MED Science, Osaka, Japan) was used to record the electrical activity of neuronal networks. Neurons were plated at a density of 5000–6000 cells/mm^2^ on a MED probe (Cat#: MED-R530A; carbon nanotube electrodes, 64 channels; interelectrode spacing, 250 μm). At 4 days after seeding, cytosine arabinoside (5 μM) was added to the growth medium containing the neurons to prevent glial proliferation. The field potentials in autonomously reconstructed neuronal networks were recorded at 37°C in 5% CO_2_ and 95% air with saturated humidity throughout the experiment. The recorded spikes were automatically analyzed using BURSTSCOPE software (Alpha MED Science). Briefly, the mean noise level was calculated for each individual channel, and a threshold was set at 5 standard deviations from the mean. A spike was then detected if the signal surpassed this threshold. Channels were considered to be active if the spike rate was at least 0.1 Hz. Detected spikes were classified by their amplitude versus decay time distributions using k-means cluster analysis with original preprocessing and automatically classified into single units. A burst was defined as the occurrence of a minimum of 4 spikes not more than 75 milliseconds (msec) apart from each other. The minimum interburst interval was set to 100 msec. Synchronized bursts were calculated from the array-wide spike detection rate (ASDR). The ASDR was measured as the total number of spikes across the entire array in each second of recording averaged over the entire recording.

### Statistical analysis

Statistical analysis was performed after the Shapiro–Wilk normality test with SPSS software version 20 (SPSS Inc., Chicago, IL) and in Microsoft Excel (Microsoft, Redmond, WA, USA), followed by the analysis of normal data (n=20 or more) by Student’s t-test and one-way analysis of variance (ANOVA). The Mann-Whitney U test was employed for nonparametric data (n=less than 20). This study did not involve randomization or blinding. Sample sizes were determined based on the expected effect size derived from pilot experiments. The study was not preregistered.

## Results

### Identification of the Prickle2 interactome with mass spectrometry

**Fig. 1:**
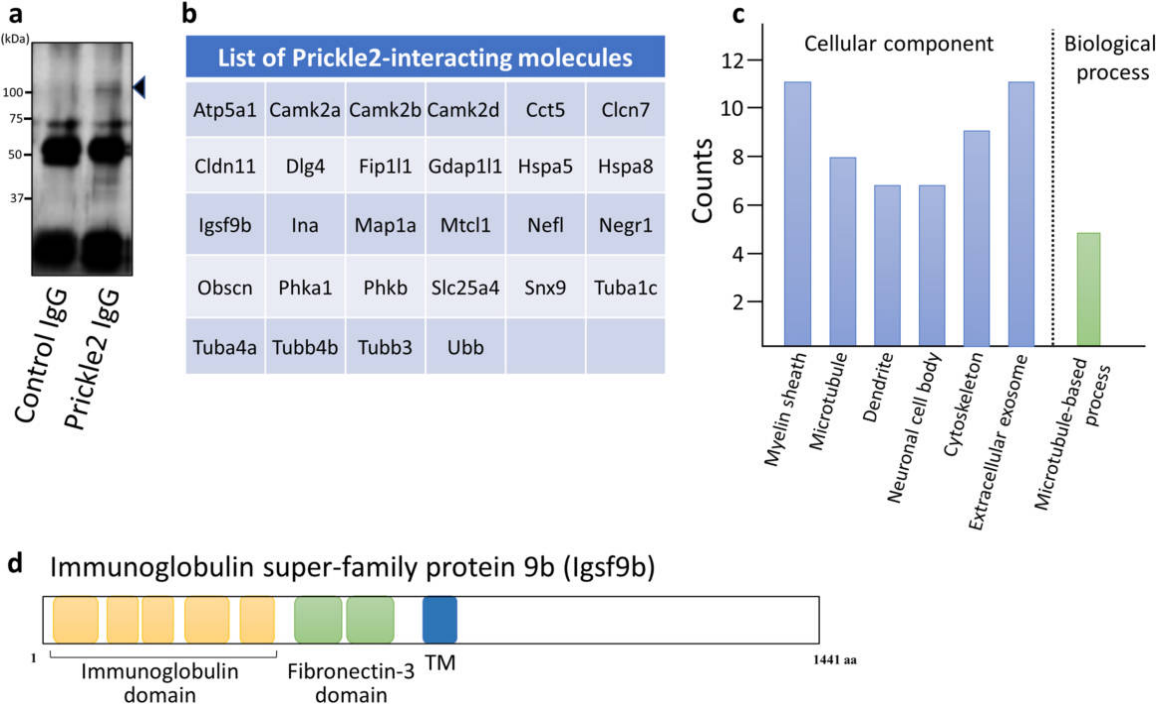
Identification of Prickle2-interacting molecules. (a) Prickle2 immunoprecipitates from whole mouse brain lysates were analyzed by SDS-PAGE, followed by immunoblotting with anti-Prickle2 antibody. The arrowhead indicates the Prickle2 protein. (b) Summary of Prickle2-interacting molecules. We defined molecules identified by mass spectrometry analysis of Prickle2 immunoprecipitates as promising Prickle2 interactors. (c) Gene ontology analysis of the Prickle2 interactome. The proteins identified by mass spectrometry analysis were classified according to the gene ontology (GO) groups, cellular component and biological process (indicated in the X-axis). The Y-axis indicates the total number of proteins categorized from among the Prickle2 interactors. The p*-*values from GO annotation enrichment were less than 0.01. (d) Domain structure of immunoglobulin super-family protein 9b (Igsf9b). Igsf9b is a transmembrane protein with five immunoglobulin domains and two fibronectin-3 domains in its extracellular region.

To identify the binding partners of Prickle2 in the brain, we performed a proteomic analysis of the Prickle2 immunoprecipitate with mass spectrometry. Whole brains were dissected from C57BL/6J mice and lysed in radioimmunoprecipitation assay buffer. Endogenous Prickle2 was precipitated from the brain lysate (Fig. 1a), and the Prickle2 immunoprecipitate was then subjected to liquid chromatography-tandem mass spectrometry (LC-MS/MS) for protein identification. We identified twenty-eight potential Prickle2 interactors, including Calcium-calmodulin dependent kinases (CamK2alpha/beta/delta), PSD95/Disks large homolog 4 (Dlg4), Immunoglobulin superfamily member 9b (Igsf9b), Microtubule associated protein 1a (Map1a), Microtubule crosslinking factor 1 (Mtcl1), and α/β-tubulin subunits (Tuba1c, Tuba4a/b, Tubb3) (Fig. 1b and Supplementary Table I). Dlg4 is known as a promising binding partner of Prickle2 (Hida *et al.*, 2011; Nagaoka *et al.*, 2015), suggesting that immunoprecipitation with an anti-Prickle2 antibody isolated components of the Prickle2 interactome. Gene ontology analysis of the identified molecules revealed that the Prickle2 interactome is associated with the myelin sheath, microtubules, and microtubule-based processes (Fig. 1c). Among the Prickle2 interactors, Igsf9b is a transmembrane protein with immunoglobulin and fibronectin-3 domains (Fig. 1d). The dysregulation of Igsf9 family proteins has been implicated in the pathology of neurological diseases, including seizures, major depression, and schizophrenia (Mishra *et al.*, 2014; Schizophrenia Working Group of the Psychiatric Genomics, 2014; Shyn *et al.*, 2011). Here, we focused on the physiological meaning of the interaction between Prickle2 and Igsf9b because *Drosophila* Prickle and Igsf9 regulate forward transport to axons (Ehaideb *et al.*, 2014; Shaw *et al.*, 2019).

### Interaction between Prickle2 and Igsf9b *in vivo*

**Fig. 2:**
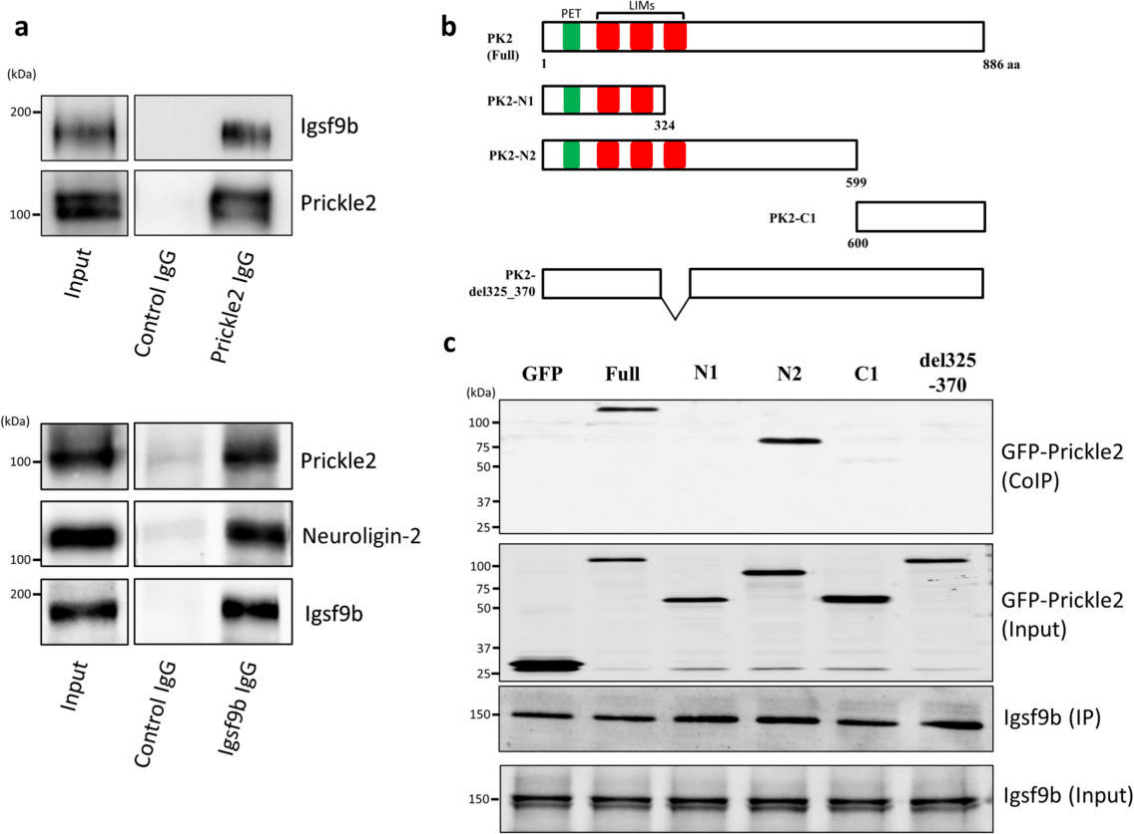
Prickle2 interacted with Igsf9b *in vivo*. (a) *In vivo* interaction between Prickle2 and Igsf9b. Proteins immunoprecipitated with anti-Prickle2 or anti-Igsf9b antibody and assay inputs were analyzed by immunoblotting with specific antibodies against the indicated proteins. Aliquots of the original samples (10% input) and eluted samples (30%) were subjected to SDS-PAGE followed by immunoblotting. (b) Full-length Prickle2 (PK2-Full) and Prickle2 deletion mutant constructs (PK2-N1, PK2-N2, PK2-C1 and del325-370). Numbers refer to the amino acid positions in mouse Prickle2. Prickle2 has a PET domain (green rectangle) and three LIM domains (LIMs, red rectangles). (c) Interaction of Igsf9b with full-length Prickle2 and its fragments in HEK293 cells. Cells transfected with Igsf9b and the indicated Prickle2 constructs were lysed in RIPA buffer. The cell lysates were precipitated with anti-Igsf9b antibody (IP). Coprecipitated proteins were analyzed by immunoblotting with anti-GFP antibody (CoIP). Aliquots of the original samples (2% input) and eluted samples (15%) were subjected to SDS-PAGE followed by immunoblotting.

To investigate the binding of Prickle2 to Igsf9b *in vivo*, we performed an immunoprecipitation assay with specific antibodies against Prickle2 and Igsf9b. When Prickle2 was precipitated from the brain lysate with the validated anti-Prickle2 antibody (Hida *et al.*, 2011), Igsf9b was detected in the Prickle2 immunoprecipitate (Fig. 2a). In addition, the coprecipitation of Prickle2 and Igsf9b was confirmed when Igsf9b was precipitated from the brain lysate with a specific antibody (Fig. 2a). To validate the components of the Igsf9b immunoprecipitate, we also confirmed the coprecipitation of Igsf9b and Neuroligin-2, which was identified as a binding partner of Igsf9b (Woo *et al.*, 2013) (Fig. 2a). These results indicated the *in vivo* interaction between Prickle2 and Igsf9b. We next constructed several GFP-tagged Prickle2 to examine the underlying Prickle2 domain responsible for its association with Igsf9b. Full-length Igsf9b and Prickle2 deletion fragments were employed in the binding assay (Fig. 2b). The N2 fragment of Prickle2 (PK2-N2, amino acids [aa] 1–599), as well as full-length Prickle2, bound to Igsf9b, whereas PK2-N1 (aa 1–324) and PK2-C1 (aa 600–886) did not bind to Igsf9b (Fig. 2c). The Prickle2 mutant lacking the third LIM domain of Prickle2 (PK2-del325_370) lost the ability to interact with Igsf9b (Fig. 2c). These results indicated that the third LIM domain of Prickle2 is indispensable for the Prickle2/Igsf9b interaction.

### Prickle2 is required for the localization of Igsf9b in hippocampal neurons

**Fig. 3:**
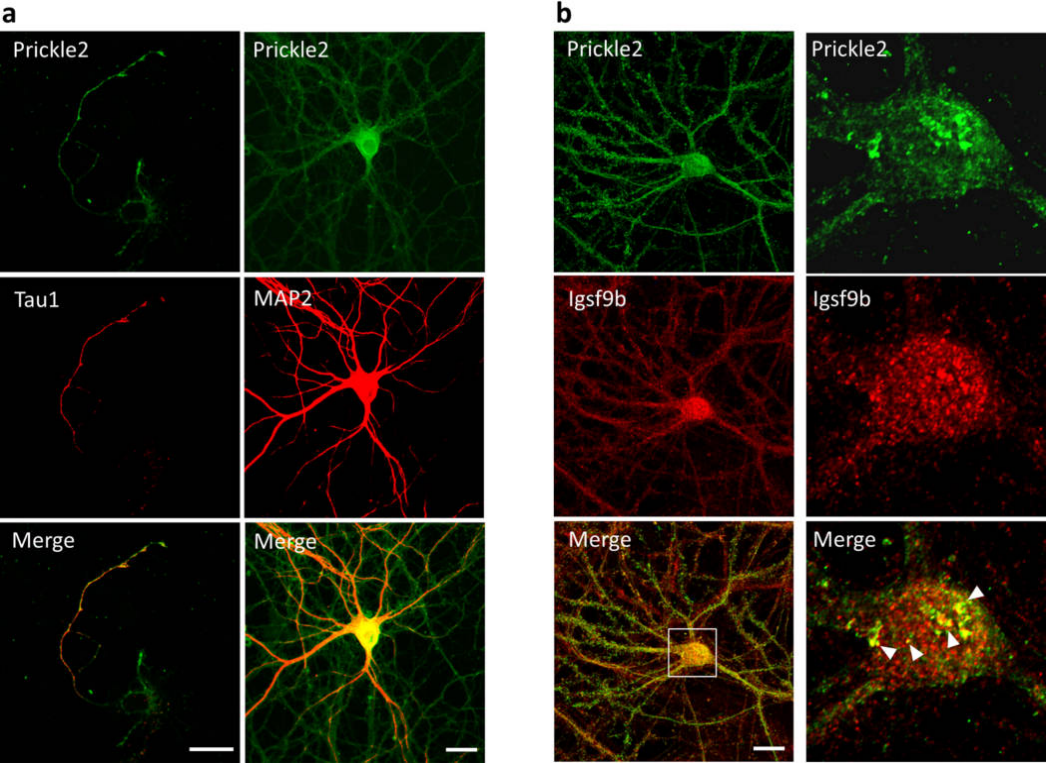
Prickle2 colocalized with Igsf9b in hippocampal neurons. (a) Prickle2 distribution in cultured mouse hippocampal neurons. In nascent, 3 DIV neurons, a robust Prickle2 immunosignal was detected at the developing axons, which were labeled by Tau (left column). In older, 14 DIV neurons, Prickle2 was distributed at all of the neuronal processes with or without MAP2 signals (right column). Bars, 20 μm. (b) Colocalization of Prickle2 with Igsf9b in neuronal cells. Cultured neurons at 21 DIV were immunostained with anti-Prickle2 and anti-Igsf9b antibodies. Prickle2 was distributed in axon-like processes and the somatodendrites of cultured neurons (left column). Highly magnified images (right column) show the colocalization of Igsf9b and Prickle2 in the soma of the cultured neuron. The arrowheads indicate the overlap of punctate structures consisting of both Igsf9b and Prickle2. Bars, 20 μm.

**Fig. 4:**
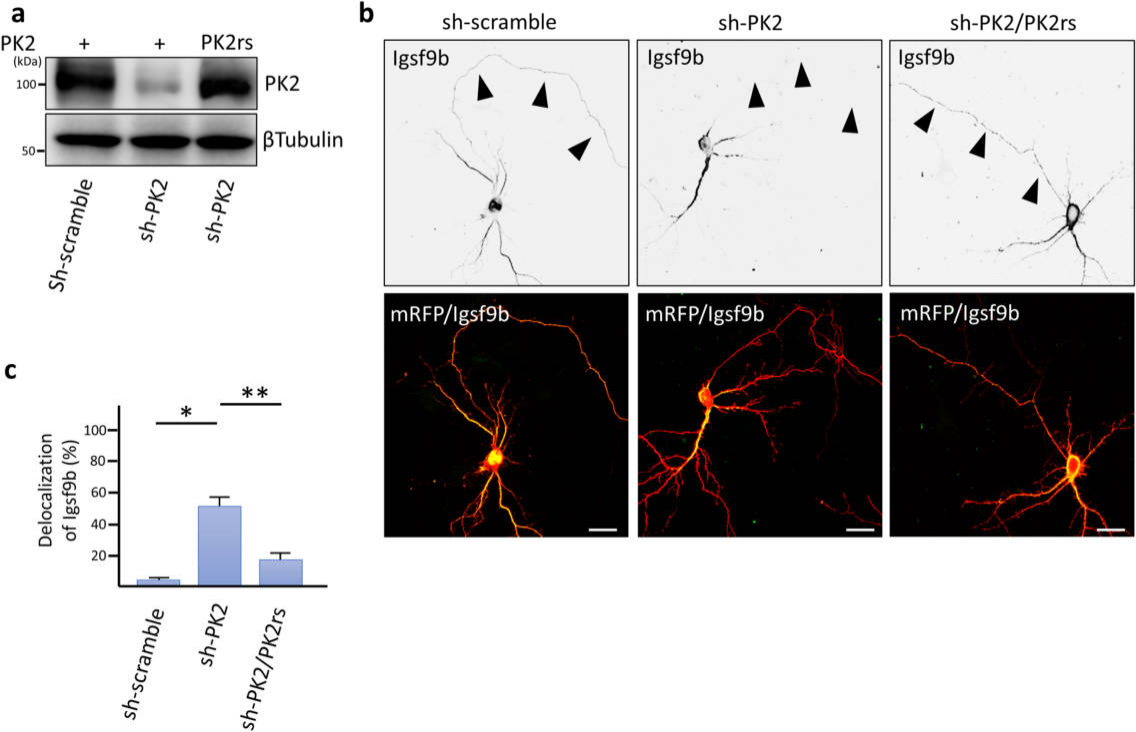
Prickle2 knockdown abolished the localization of Igsf9b. (a) Knockdown of mouse Prickle2 with RNA interference (RNAi). Gene silencing of target proteins by RNAi was validated by cotransfection of HEK293 cells with pSUPER RNAi vectors carrying a scrambled sequence (sh-scramble) or a sequence targeting Prickle2 (sh-PK2) and vector containing the HA-Prickle2 construct. For the rescue experiment, we cotransfected an RNAi-resistant Prickle2 mutant, PK2rs, in the knockdown neurons. (b) Effect of Prickle2 knockdown on Igsf9b localization. Hippocampal neurons at 11 DIV were transfected with sh-scramble or sh-PK2 and mRFP. At 3 days after transfection, the transfected neurons were fixed and observed. For the rescue experiment, we cotransfected PK2rs into the knockdown neurons. Upper panels contain grayscale images showing Igsf9b immunofluorescence. Lower panels contain merged images from a neuron immunostained with specific antibodies against Igsf9b (green) and mRFP (red). Arrowheads indicate the axonal processes of the transfected neurons. Bars, 20 μm. (c) Quantification of the delocalization of Igsf9b. The localization of Igsf9b was validated based on the Igsf9b immunosignal at the distal neurites (more than 80 μm away from the soma). Data are expressed as the mean±SE. Asterisks indicate significant differences (* *P*<0.05, compared with sh-scramble; and ** *P*<0.05, compared with sh-PK2, n=45 for each experiment, ANOVA).

We examined the subcellular distribution of Prickle2 in cultured mouse hippocampal neurons. In a nascent neuron, Prickle2 was localized at the developing axon, which was labeled by Tau1 (Fig. 3a). In the older neurons, Prickle2 appeared to be located in the axonal and dendritic processes because an immunosignal for Prickle2 was detected in neurites labeled with or without the dendritic marker MAP2 (Fig. 3a). To evaluate the region where Prickle2 and Igsf9b associate at the intracellular level, we performed an immunofluorescence study with specific antibodies against Prickle2 and Igsf9b. Igsf9b was distributed in the axons and somatodendrites of mature hippocampal neurons and partly colocalized with Prickle2 in the soma of these neurons (Fig. 3b). Igsf9b immunofluorescence was not detected in nascent neurons (data not shown), as suggested in a previous report (Woo *et al.*, 2013). To examine the cellular process associated with Prickle2, we performed immunohistochemical studies with molecular markers for organelles. Prickle2 colocalized with the transport regulators such as Rab5 and Rab8 and, to a lesser extent, Rab3, the endoplasmic reticulum marker Bip and the lysosome marker Lamp1 (Fig. S1a and b). Given the colocalization of Prickle2 with Rab5 and Rab8 (Kiral *et al.*, 2018; Ng and Tang, 2008), we postulated that Prickle2 regulates the trafficking of vesicular compartments containing Igsf9b. We employed RNA interference (RNAi) to evaluate whether Prickle2 is necessary for Igsf9b localization. The transfection of an RNAi vector encoding a short hairpin RNA targeting Prickle2 (sh-PK2) significantly suppressed expression of the *Prickle2* transgene and endogenous Prickle2 (Fig. 4a, Fig. S2a and b). The localization of Igsf9b was significantly different from Prickle2-knockdown neurons and control neurons transfected with sh-scramble (Fig. 4b, c and Fig. S2c). To validate the specificity of Prickle2 knockdown, we constructed an RNAi-resistant Prickle2 mutant (PK2rs) and then performed a rescue experiment in Prickle2-knockdown neurons with PK2rs. The impairment of Igsf9b localization in Prickle2-knockdown neurons was restored by the expression of PK2rs (Fig. 4b and c). To further investigate the effect of Prickle2 knockdown on the subcellular distribution of Igsf9b, we measured the fluorescence intensity of Igsf9b in the axons and dendrites of wild-type and Prickle2-knockdown neurons (Fig. S3a and b). Prickle2 knockdown caused a defect in Igsf9b localization in axons and, to a lesser extent, a similar defect in dendrites (Fig. S3a and b). These results suggest that Prickle2 regulates the transport of Igsf9b to the neurites of hippocampal neurons.

### Prickle2 collaborates with Igsf9b to regulate AIS cytoarchitecture

**Fig. 5:**
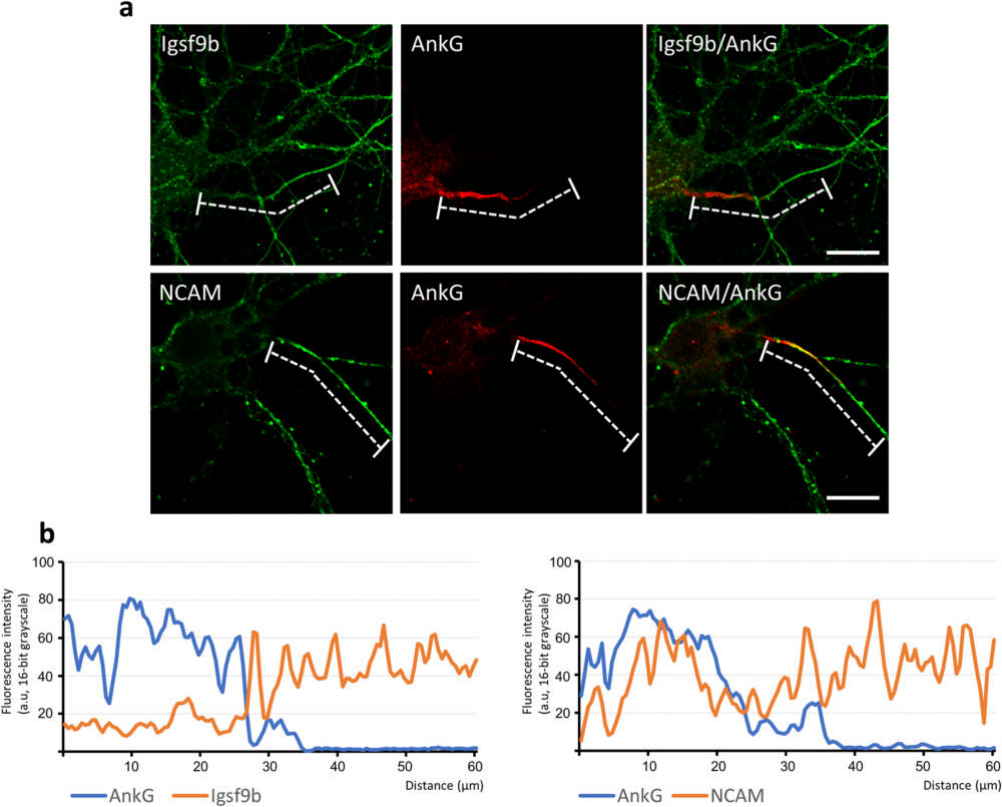
Inverse localization of Igsf9b to the axon initial segment. (a) Comparative distribution of Igsf9b and AnkG or NCAM in axons. Hippocampal neurons at 14 DIV were immunostained with antibodies specific for the indicated proteins. Dashed lines indicate the areas measured in the line-scanning study in panel b. Bars, 20 μm. (b) Line scanning of the fluorescence intensities of AnkG, Igsf9b and NCAM. The original images of each protein were converted to grayscale images. The X-axis indicates the length of the dashed lines. The Y-axis indicates the fluorescence intensities as grayscale values (16 bits, ranging from 0 to 255 bits).

**Fig. 6:**
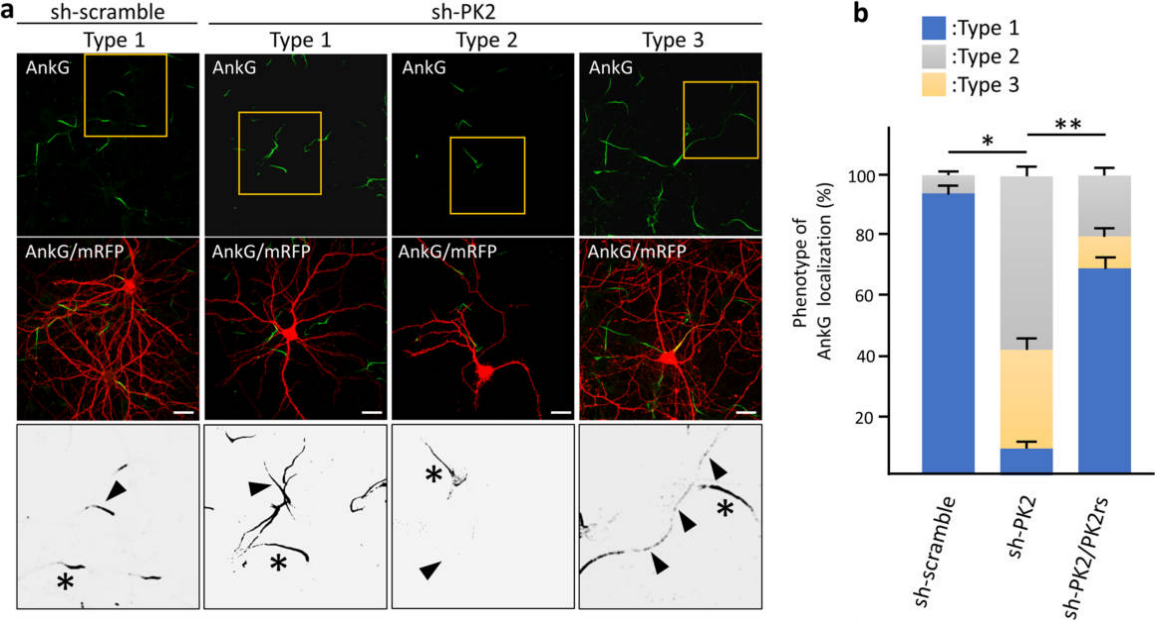
Knockdown of Prickle2 affected the AnkG distribution at the proximal region of axons. (a) Effect of Prickle2 knockdown on the localization of AnkG. Representative images of the three patterns (types 1 to 3) of AnkG distribution are shown. Hippocampal neurons at 11 DIV were transfected with sh-scramble or sh-PK2 and mRFP. At 3 days after transfection, the transfected neurons were immunostained with specific antibodies against AnkG (green) and mRFP (red). For the rescue experiment, we cotransfected PK2rs into the knockdown neurons. Upper and middle panels show AnkG immunofluorescence images and these images merged with mRFP immunofluorescence images. Highly magnified enlarged images corresponding to the yellow squares in the upper panels were converted to grayscale images and are shown in the lower panels. Arrowheads and asterisks indicate the AnkG distribution in the shRNA-transfected or nontranfected neurons, respectively. Bars, 20 μm. (b) Quantification of the AnkG distribution patterns. The phenotypes in shRNA-transfected neurons were categorized according to the AnkG immunosignal (type 1, normal AnkG labeling in the proximal region; type 2, no AnkG labeling; type 3, discontinuous labeling indicative of fragmentation). Data are expressed as the mean±SE. Asterisks indicate significant differences (* *P*<0.05, compared with sh-scramble; and ** *P*<0.05, compared with sh-PK2, n=45 for each experiment, ANOVA).

We addressed the axonal functions of Igsf9b to understand the physiology of Igsf9b localization mediated by Prickle2. To clearly visualize the population of Igsf9b embedded in the plasma membrane, digitonin permeabilization was used, releasing cytosolic components from cultured cells. In neurons permeabilized with digitonin, Igsf9b appeared to be enriched in the distal part of axons. We compared the distribution of Igsf9b to that of AnkyrinG (AnkG), which was localized in the part of axons proximal to the cell body. Line scanning of the fluorescence intensities of AnkG and Igsf9b showed that the AnkG fluorescence intensity peaked in the proximal part of the axon (Fig. 5a and b). In contrast, the Igsf9b fluorescence intensity gradually increased toward the distal axon. The fluorescence profile of NCAM, which is a transmembrane protein similar to Igsf9b, partly overlapped that of AnkG (Fig. 5b). These results indicated that Igsf9b is distributed along axonal processes in a pattern opposite to AnkG. Similarly, the localization of the distal axonal proteins AnkyrinB and α/βII-spectrins has been shown to be mutually exclusive with that of AnkG (Galiano *et al.*, 2012; Huang and Rasband, 2018). Distal axonal proteins control the distribution of AnkG by forming an intra-axonal boundary. Given these findings, we hypothesized that Prickle2 is involved in AnkG localization and verified this hypothesis by confirming that Prickle2 is necessary to ensure the proper localization of Igsf9b. Prickle2 knockdown caused AnkG to redistribute in different patterns (Fig. 6a and b). We divided the defective localization patterns into three phenotypes according to the impaired distribution of AnkG. Almost all the control neurons (more than 90%) showed AnkG accumulation at the proximal part of axons (Fig. 6a and b; type 1 phenotype). In contrast, AnkG was absent from the proximal part of axons in approximately 60% of prickle2-knockdown neurons (Fig. 6a and b; type 2 phenotype). The minority (approximately 30%) of Prickle2-knockdown neurons showed a discontinuous and punctate distribution of AnkG along axonal processes (Fig. 6a and b; type 3 phenotype). The expression of PK2rs restored the defective localization of AnkG (seen in phenotypes 2 and 3) in the Prickle2-knockdown neurons (Fig. 6b). To determine whether Igsf9b contributes to the spatial regulation of AnkG, we examined the effect of Igsf9b knockdown on the localization of AnkG. The Igsf9b-knockdown neurons showed defects in AnkG distribution compared to control neurons transfected with sh-scramble (Fig. S4). The similar impairment of AnkG localization in Prickle2- and Igsf9b-deficient neurons suggests that Prickle2 and Igsf9b cooperate to regulate the cytoarchitecture of AIS.

### Prickle2 hypofunction alters the network activity of cultured hippocampal neurons

**Fig. 7:**
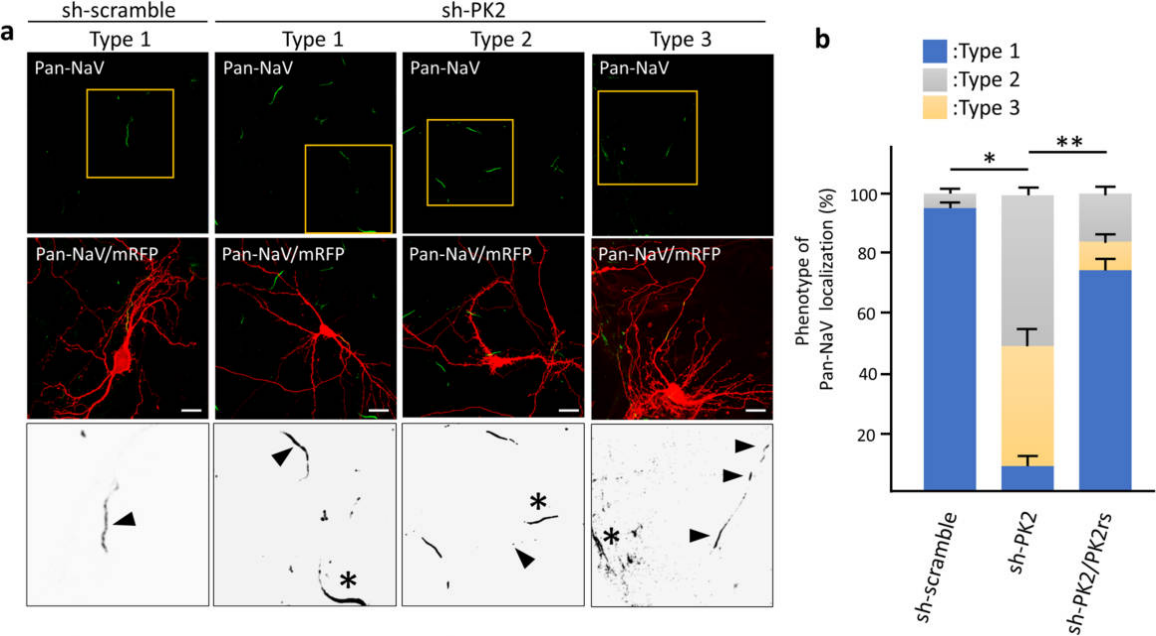
Distribution of sodium channels in PK2-KD neurons. (a) Effect of Prickle2 knockdown on the localization of voltage-gated Na^+^ channel alpha subunits (Pan-NaV). Representative images of the three Pan-NaV distribution patterns (types 1 to 3) are shown. Hippocampal neurons at 11 DIV were transfected with sh-scramble or sh-PK2 and mRFP. At 3 days after transfection, the transfected neurons were immunostained with specific antibodies against NaV (green) and mRFP (red). For the rescue experiment, we cotransfected PK2rs into knockdown neurons. Upper and middle panels show Pan-NaV immunofluorescence images and these images merged with mRFP immunofluorescence images. Highly magnified enlarged images corresponding to the yellow squares in the upper panels were converted to grayscale images and are shown in the lower panels. Arrowheads and asterisks indicate the Pan-NaV distribution in the shRNA-transfected or nontranfected neurons, respectively. Bars, 20 μm. (b) Quantification of the Pan-NaV distribution patterns. The phenotypes in shRNA-transfected neurons were categorized according to the Pan-NaV immunosignal (type 1, normal Pan-NaV labeling at the proximal region; type 2, no Pan-NaV labeling; type 3, discontinuous labeling indicative of fragmentation). Data are expressed as the mean±SE. Asterisks indicate significant differences (* *P*<0.05, compared with sh-scramble; and ** *P*<0.05, compared with sh-PK2, n=35 for each experiment, ANOVA).

AnkG functions as a scaffolding protein, facilitating the accumulation of a pan-alpha Na+ channel subunits (Pan-NaV) to modulate neuronal activity (Leterrier, 2018). We examined the effect of Prickle2 knockdown on the localization of Pan-NaV. In control neurons, Pan-NaV was distributed in the proximal part of axons, where the AIS is constructed (Fig. 7a). However, Pan-NaV in Prickle2-knockdown neurons was absent from the proximal part of axons or localized diffusely along axonal processes (Fig. 7a and b). The expression of PK2rs restored the defective localization of Pan-NaV in the Prickle2-knockdown neurons (Fig. 7b).

We next evaluated the neural network activity in Prickle2-knockdown neurons with a multielectrode array (MEA) because the redistribution of NaV would affect neural firing. Local field potentials recorded by the MEA probe usually reflect the sum of the action potentials evoked by individual neurons, thereby allowing the global activity of a neural network to be evaluated (Einevoll *et al.*, 2013). Mouse hippocampal neurons were cultured on an MEA probe with 64-channel carbon nanotube electrodes. Endogenous Prickle2 in hippocampal neurons was depleted with short interfering RNA (siRNA) because the use of siRNA enabled Prickle2 to be knocked down in most of the cultured neurons. At 3 days after siRNA transfection, the field potentials from the neurons were measured. We measured the relative spike frequency (spike number/sec), number of potential bursts, and number of synchronized bursts by calculating the field potentials recorded by the MEA probes for 3 min (Fig. S5a to c). Panel b in Fig. S5 shows representative raster plots illustrating firing in the neurons transfected with siRNAs. The raster plots showed two distinct types of firing activity in the transfected neurons: 1) randomly generated peaks of burst firing and 2) synchronously generated bursts (Fig. S5b) The neurons transfected with si-Prickle2 displayed an increased number of synchronized bursts in the 3-min recording period compared to those of the control neurons (Fig. S5b and c). Prickle2 knockdown had little effect on the spike frequency or the number of spontaneous bursts (Fig. S5c). Although we cannot provide mechanistic insight into how AIS dysregulation contributes to the alternation of firing patterns, these results indicate that Prickle2 depletion affects neural network activity.

## Discussion

### Identification of the PK2 interactome in the brain

We identified many potential Prickle2 interactors in the mouse brain. A novel partner of Prickle2, Igsf9b, was shown to regulate AIS cytoarchitecture. Gene ontology analysis revealed that the Prickle2 interactome is closely associated with microtubules and their related processes (Fig. 1c). Enrichment of these cellular processes in the Prickle2 interactome is associated with the involvement of the *Drosophila* Prickle protein in microtubule polarity and axon transport (Ehaideb *et al.*, 2014). Through a literature-based approach, we found that certain Prickle2 partners, such as Mtcl1 and Map1a, regulate microtubule (MT) organization (Kader *et al.*, 2017; Noiges *et al.*, 2002). Mtcl1 and Map1a hypofunction have been shown to cause malformation of the AIS structure via dysregulation of the MT network (Liu *et al.*, 2015; Satake *et al.*, 2017). Prickle2 can modulate microtubule organization via the interactions of MTCL1 and MAP1A. Although we have not comprehensively uncovered the molecular mechanisms of Prickle2 interactors, Prickle2 might be pleiotropically involved in the AIS cytoarchitecture through distinct protein-protein interactions. Ultimately, mass spectrometry of the Prickle2 immunoprecipitate revealed that AIS regulators converge in the Prickle2 interactome.

### Implication of the dysregulated AIS in the alternation of excitability and circuit activity

Although the regulatory mechanism underlying the AIS location remains to be fully understood, it has been proposed that the distal axonal cytoskeleton, which is composed of AnkyrinB and α/βII-spectrins, functions as the intra-axonal boundary that specifies the AIS area (Galiano *et al.*, 2012; Huang and Rasband, 2018). These cytoskeletal proteins localize at the distal part of axons to spatially restrict AIS proteins such as AnkG and some sodium channels. Igsf9b was distributed at the distal region of the axon, in accordance with the intra-axonal barrier model. Knockdown experiments demonstrated that Prickle2 is required for Igsf9b localization and that Prickle2 and Igsf9b control positioning of the AnkG. We found the colocalization of Prickle2 with Rab5 and Rab8 in the immunofluorescence study. Rab5 and Rab8 control early endosome formation and axonal transport in neurons, respectively (Ng and Tang, 2008). Given the colocalization of Prickle2 with Rab5 and Rab8, we suggest that Prickle2 is involved in the transport and recycling of vesicular compartments. These findings suggest that Prickle2 controls the AnkG distribution by modulating the proper localization of Igsf9b (Fig. S6). An intriguing question is how Prickle2 regulates the transport of Igsf9b to the distal end of neurites. This issue would be beyond the scope of this study. We attribute the dissociation of AIS cluster caused by the gene silencing against *Prickle2* and *Igsf9b* to the lack of intra-axonal boundary. Since Igsf9b is expressed in mature neurons (Woo *et al.*, 2013), Igsf9b might function in AIS plasticity but not AIS formation. Changes in the structure and distribution of the AIS can have important consequences for neuronal excitability, as the AIS fine-tunes neuronal output modulation (Hamada *et al.*, 2016; Yamada and Kuba, 2016). Moreover, the structural properties of the AIS, such as its length and location relative to the soma, change in an activity-dependent manner (Yamada and Kuba, 2016). Hence, spatial regulation of the AIS is crucial for the homeostatic control of neuronal excitability. To address the barrier function of axonal Igsf9b for AIS plasticity, we need to evaluate the interaction between Prickle2 and Igsf9b upon neural activity.

### Association between the AIS cytoarchitecture and comorbidity in ASD and epilepsy

The *ANK3* gene was identified as a promising susceptibility gene in a wide range of whole-genome sequencing studies of ASD patients (Bi *et al.*, 2012; Iqbal *et al.*, 2013; Kloth *et al.*, 2017; Shi *et al.*, 2013). *ANK3* encodes a giant splice variant of Ankyrin-G (AnkG) that is located at the nodes of Ranvier and AIS. In a mouse model of AnkG, AnkG disruption caused convulsive seizures (Zhou *et al.*, 1998). Seizure generation was reproduced by the imbalance of excitatory and inhibitory activities triggered by AnkG depletion (Lopez *et al.*, 2017). These reports suggest that AnkG is involved in the comorbidity between ASD and epilepsy. SCN2A encodes a voltage-gated sodium channel subunit with a known role in the AIS and is robustly associated with ASD (Ben-Shalom *et al.*, 2017; Buxbaum *et al.*, 2012). Mutations in the SCN2A gene have been identified in families affected by neonatal and infantile seizures (Heron *et al.*, 2002; Scalmani *et al.*, 2006; Sugawara *et al.*, 2001). *Scn2a* heterozygous mice showed absence-like seizures and mild social behavior impairment (Ogiwara *et al.*, 2018; Tatsukawa *et al.*, 2019). Haploinsufficiency and polymorphisms of *ANK3* and *SCN2A* in patients with ASD and epilepsy imply that AIS dysfunction is involved in the comorbidity of these diseases. In this study, we found that Prickle2 knockdown impaired the localization of AnkG and voltage-gated sodium channels (Fig. 6 and Fig. 7), thereby affecting neural network activity (Fig. S5). The molecular mechanism by which Prickle2 underlies AIS dysregulation provides a positive clue for understanding shared pathogenic signaling between ASD and epilepsy.

## Abbreviations

ASD: autism spectrum disorder
HA: hemagglutinin
GST: glutathione S-transferase
GFP: green fluorescent protein
RIPA: radioimmunoprecipitation assay
SDS: sodium dodecyl sulfate
AnkG: Ankyrin-G
NCAM: neural cell adhesion molecule
NaVs: voltage-gated Na+ channels
SDS-PAGE: sodium dodecyl sulfate-polyacrylamide gel electrophoresis
RNAi: RNA interference
shRNA: short hairpin RNA
siRNA: small interfering RNA
