# Supplementary figures and images for: Prickle2 and Igsf9b Coordinately Regulate the Cytoarchitecture of the Axon Initial Segment
Source: Cell Struct Funct. 2020 Jul 8;45(2):143–54. doi: 10.1247/csf.20028 (PMC10511046; doi:10.1247/csf.20028)

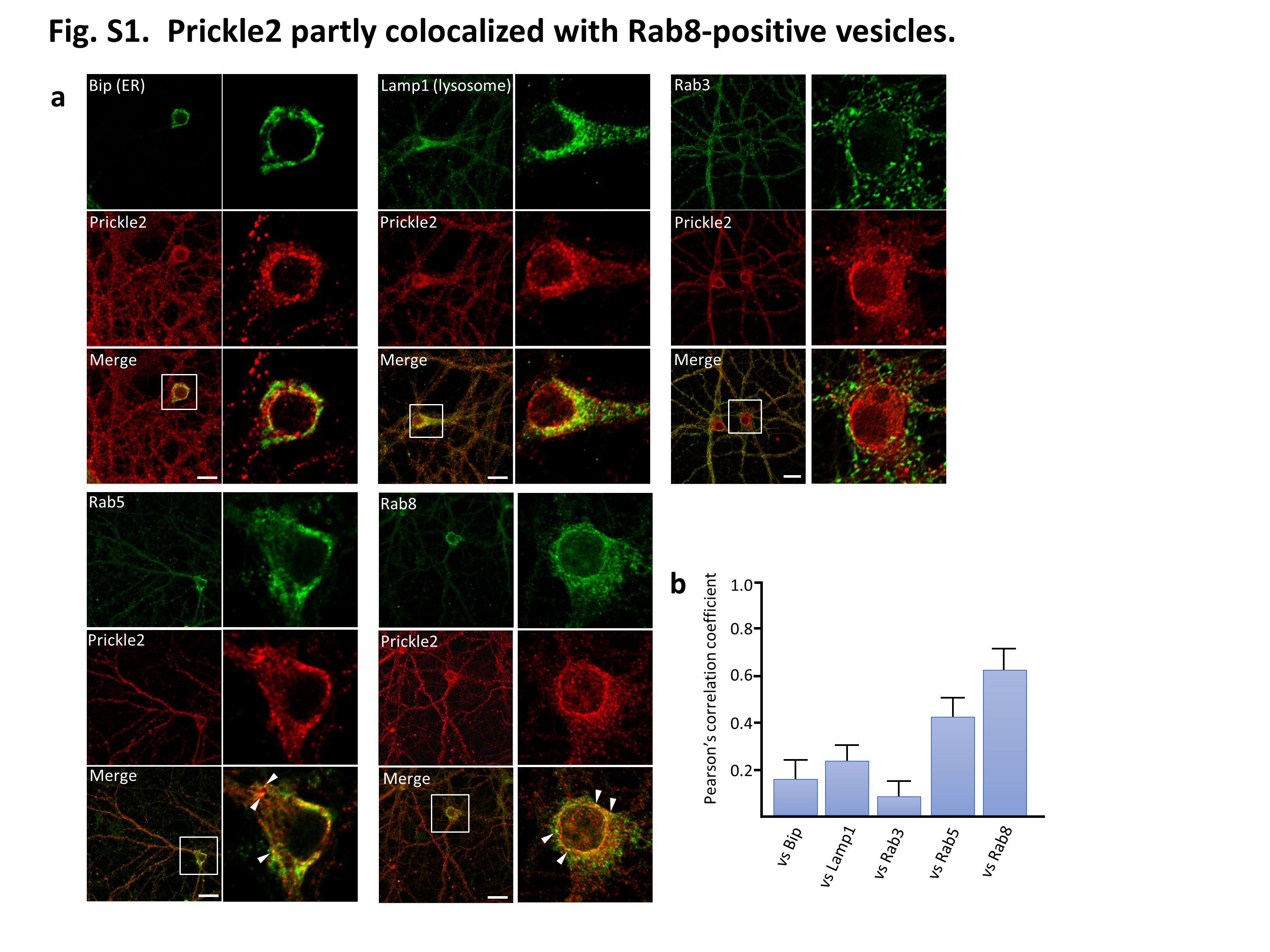

Supplement: Supplementary file 1 — Fig. S1 [file csf_45_20028_1.tif]

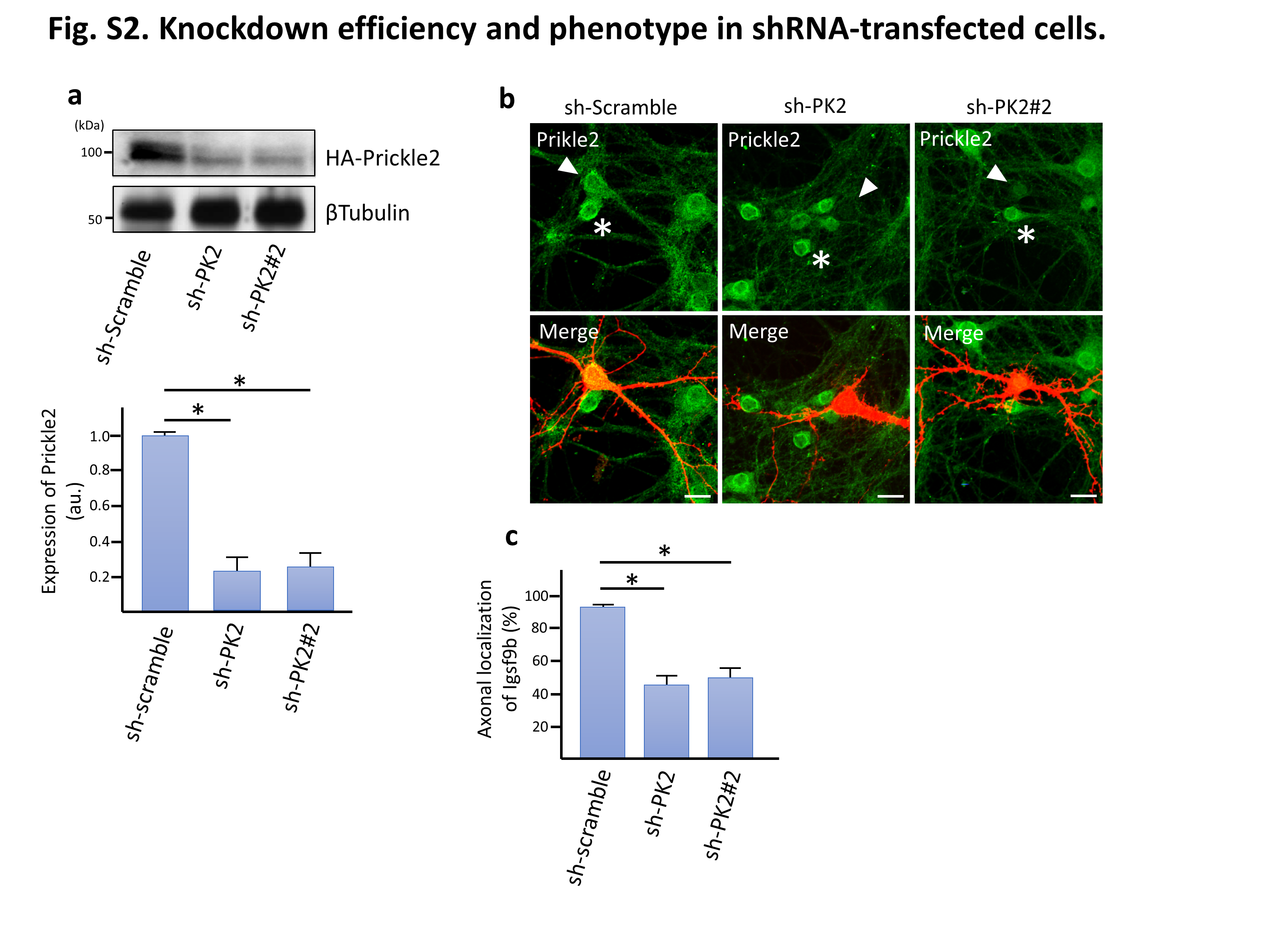

Supplement: Supplementary file 2 — Fig. S2 [file csf_45_20028_2.tif]

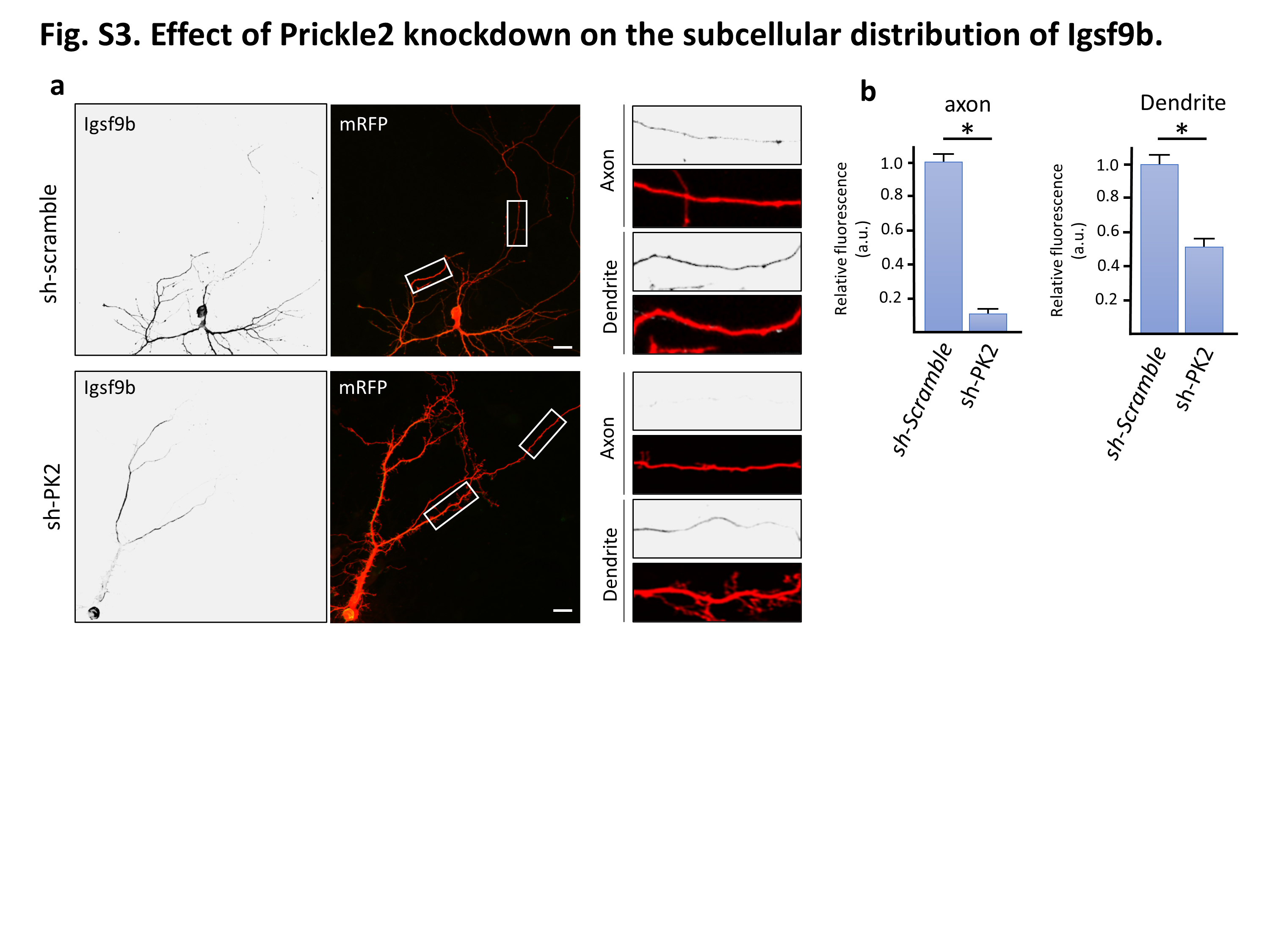

Supplement: Supplementary file 3 — Fig. S3 [file csf_45_20028_3.tif]

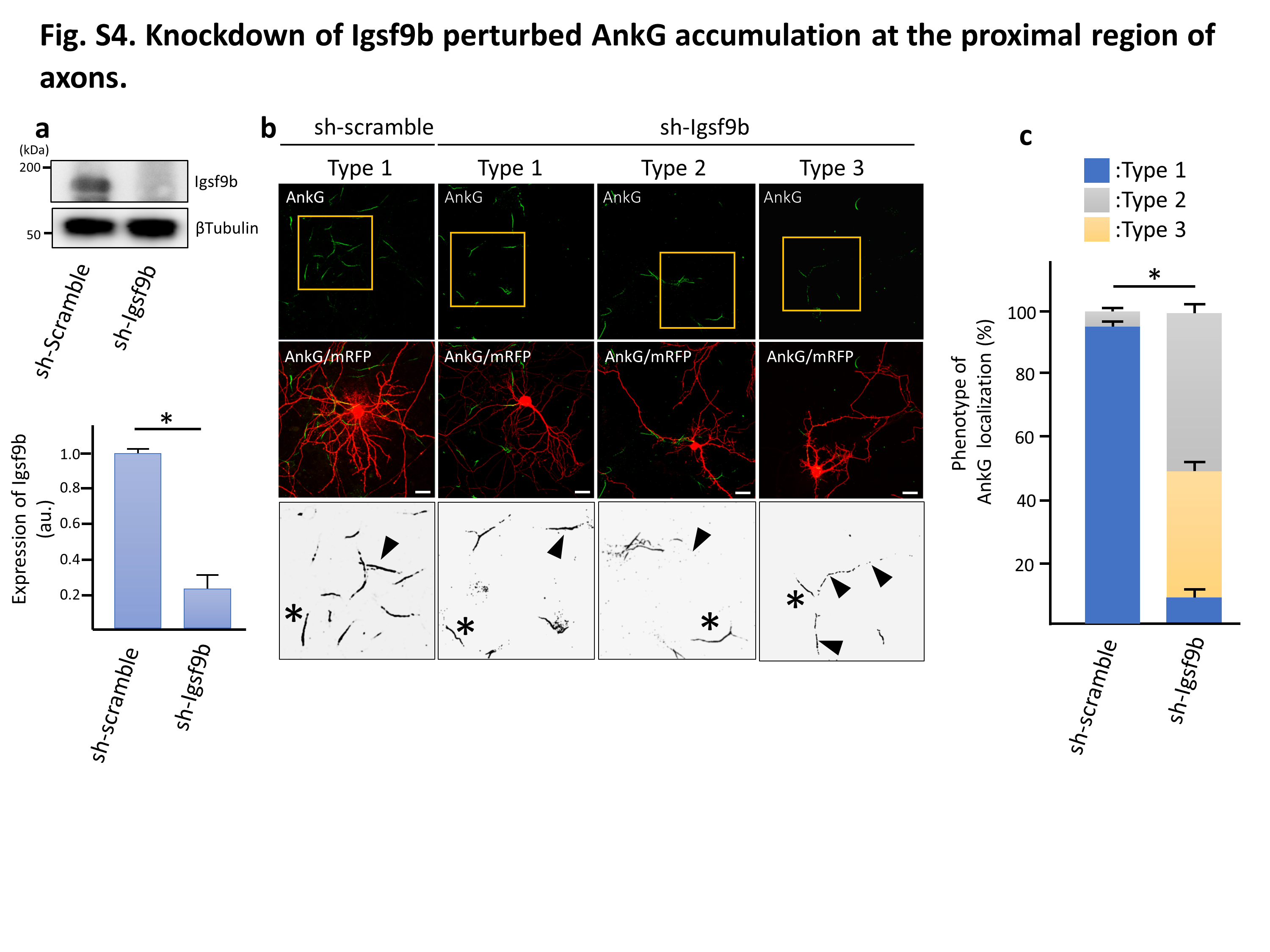

Supplement: Supplementary file 4 — Fig. S4 [file csf_45_20028_4.tif]

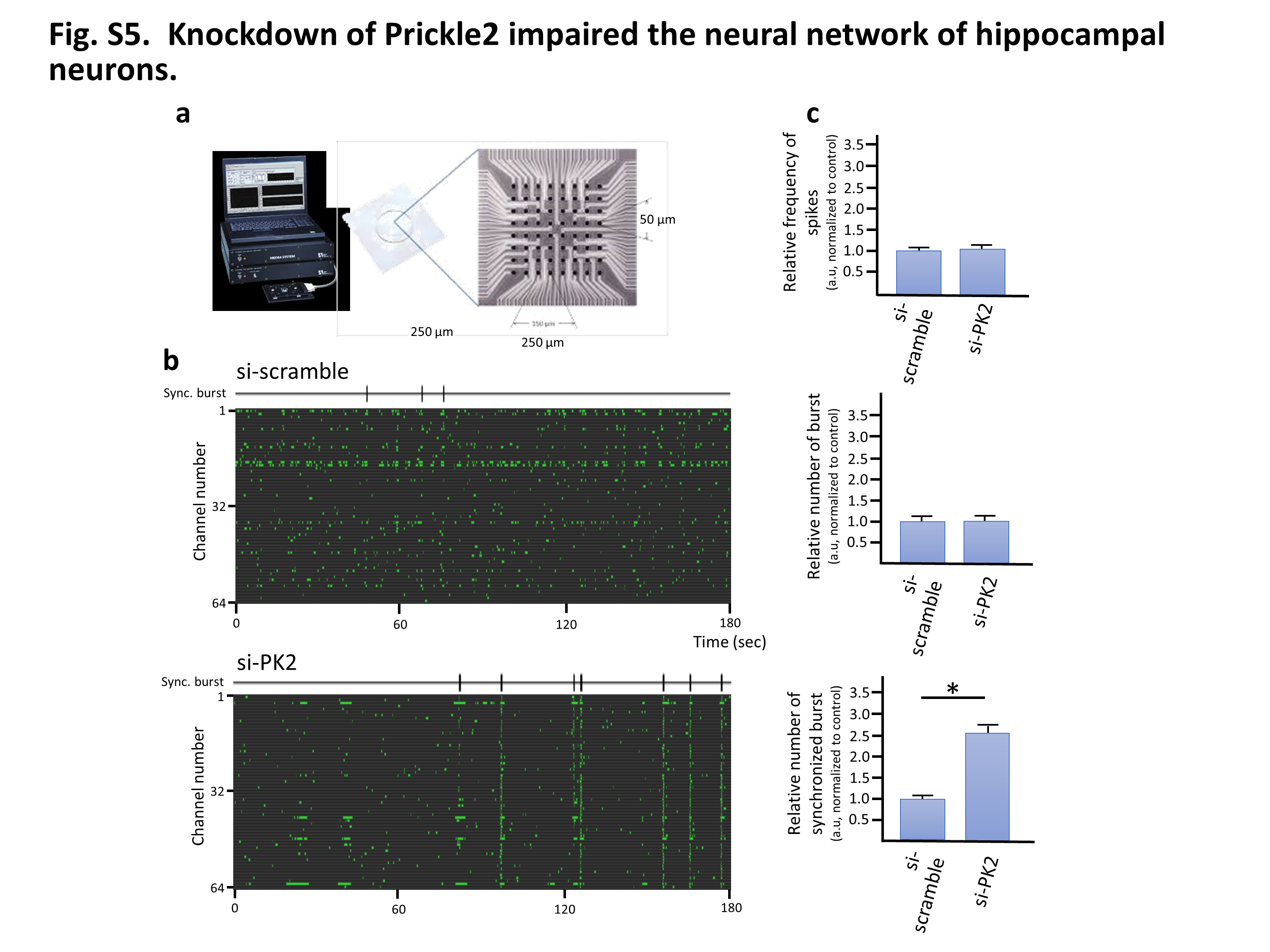

Supplement: Supplementary file 5 — Fig. S5 [file csf_45_20028_5.tif]

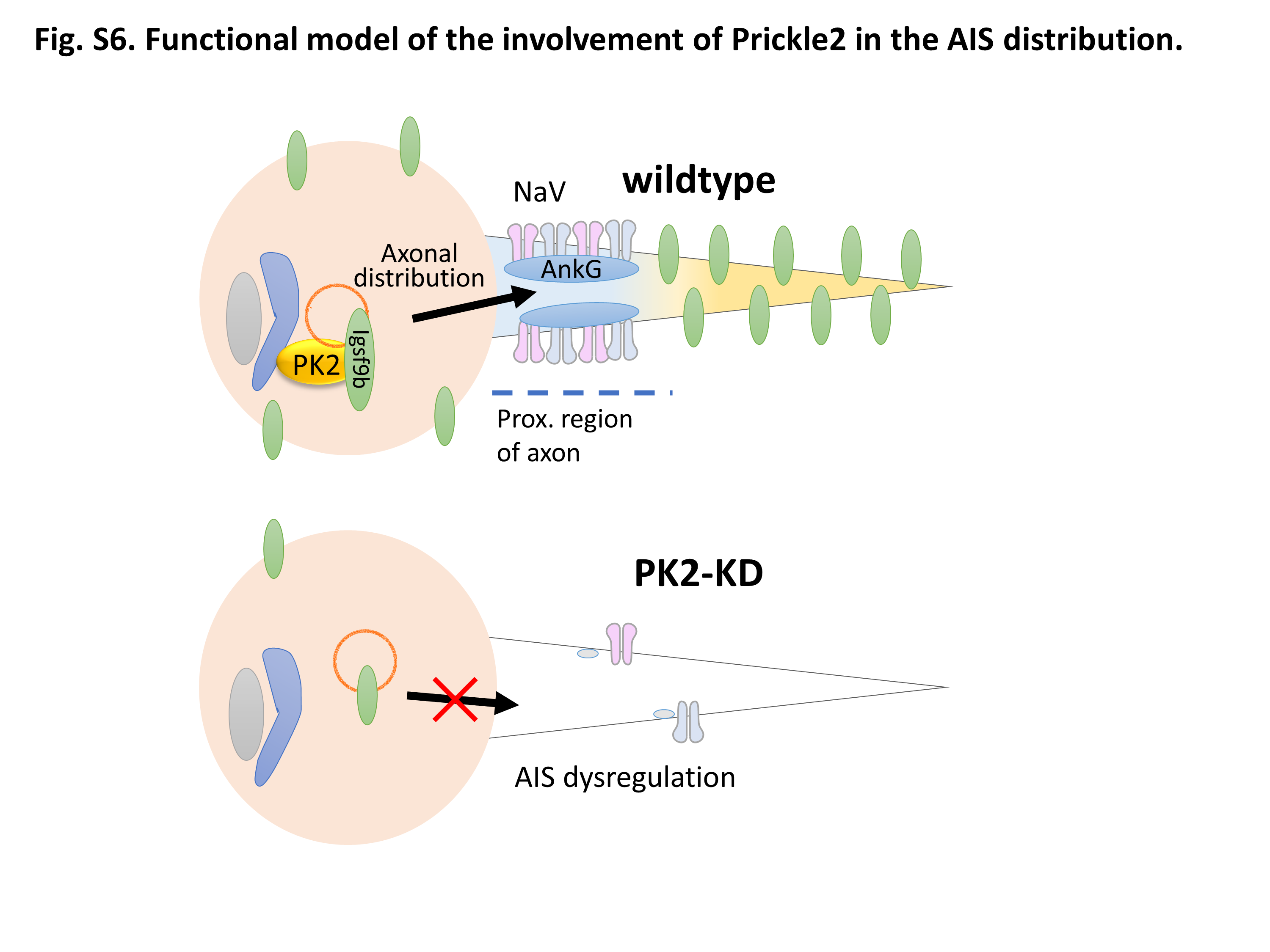

Supplement: Supplementary file 6 — Fig. S6 [file csf_45_20028_6.tif]
